# Adsorption of Toll-Like Receptor 4 Agonist to Alum-Based Tetanus Toxoid Vaccine Dampens Pro-T Helper 2 Activities and Enhances Antibody Responses

**DOI:** 10.1155/2015/280238

**Published:** 2015-08-25

**Authors:** Juliana Bortolatto, Luciana Mirotti, Dunia Rodriguez, Eliane Gomes, Momtchilo Russo

**Affiliations:** Department of Immunology, Institute of Biomedical Science, University of São Paulo, 05508-000 São Paulo, SP, Brazil

## Abstract

Aluminum salts gels (alum) are TLR-independent adjuvants and have been used to boost antibody responses in alum-based vaccines such as diphtheria, pertussis, and tetanus toxoid (DPT) triple vaccine. However, the pro-Th2 activity of alum-based vaccine formulations has not been fully appreciated. Here we found that alum-based tetanus toxoid (TT) vaccine was biased toward a Th-2 profile as shown by TT-induced airway eosinophilic inflammation, type 2 cytokine production, and high levels of IgE anaphylactic antibodies. The adsorption into alum of prototypic TLR4 agonists such as lipopolysaccharides (LPS) derived from *Escherichia coli* consistently dampened TT-induced Th2 activities without inducing IFN*γ* or Th1-like responses in the lung. Conversely, adsorption of monophosphoryl lipid A (MPLA) extracted from *Salmonella minnesota*, which is a TIR-domain-containing adapter-inducing interferon-*β*- (TRIF-) biased TLR4 agonist, was less effective in decreasing Th-2 responses. Importantly, in a situation with antigenic competition (OVA plus TT), TT-specific IgG1 or IgG2a was decreased compared with TT sensitization. Notably, LPS increased the production of IgG1 and IgG2a TT-specific antibodies. In conclusion, the addition of LPS induces a more robust IgG1 and IgG2a TT-specific antibody production and concomitantly decreases Th2-cellular and humoral responses, indicating a potential use of alum/TLR-based vaccines.

## 1. Introduction

Adjuvants fall into two major functional groups based on whether their immune activity is dependent or nor not on Toll-like receptor (TLR) signaling [1–4]. Both groups can be defined as compounds that potentiate humoral and cellular adaptive immune responses to specific antigens. The rationale for using TLR-based adjuvants is supported by their capacity to mimic natural ligands released during infections such as those derived from bacterial walls [5, 6] or from endocytosed nucleic acids. As such, they induce effector Th1/Th17 cells required to protect the host from infections [7, 8]. The molecular mechanisms of TLR-based adjuvants ultimately involve signaling thorough MyD88 or Toll/IL-1R domain-containing adaptor inducing IFN-*β*-deficient (TRIF) adapter molecules [7, 9]. The augmentation of immune responses by TLR-based vaccine could be due to stimulation of dendritic cells or B cells, which are known to express TLRs [10]. In contrast, aluminum salts (alum) and their gel variations are TLR-independent adjuvants and have also been empirically used to boost antibody responses in alum-based vaccines such as triple vaccine DPT (diphtheria, pertussis, and tetanus), human papillomavirus, and hepatitis vaccines [11, 12]. However, the pro-Th2 activity of alum-based vaccine formulations has not been fully appreciated. In addition, controversy exists regarding the requirement of TLR signaling for the antibody-enhancing effects of adjuvants [13, 14].

It is now apparent that adjuvants act in the early stages of an immune response, activating innate cells that, in turn, release cytokines and chemokines to prime naïve CD4+ T cells towards effector functions [8]. A number of murine studies have demonstrated that immunization with alum provokes strong antigen-induced Th2 responses, characterized by tissue/organ eosinophilic inflammation and elevated anaphylactic IgE levels [15]. For this reason, alum has been considered a pro-Th2 adjuvant and has been classically used in ovalbumin (OVA) asthma models [16]. However, the pro-Th2 effects of alum, such as promotion of IgE response, might be detrimental in human immunization. Indeed, though being rare, anaphylactic reactions have been reported after DPT vaccine [17].

We have previously shown in a murine OVA model that absorption of bacterial lipopolysaccharide (LPS), a prototypic TLR4 agonist, to alum inhibited the development of OVA-induced Th2 responses in a dose-dependent manner. This inhibition was via MyD88, but not TRIF pathway, and did not induce IFN*γ* or Th1-like responses in the lung [18, 19]. Therefore, it appears that combining opposing adjuvants blocks the appearance of polarized effector Th2 or Th1 cells. This situation might be particularly important for the development of vaccines aiming to potentiate antibody responses for the elicitation of neutralizing antibodies.

In order to extend these observations and as a proof of concept, we tested the effect of LPS absorption to alum-based vaccine with a deactivated tetanus toxin (TT). Because LPS signals via MyD88 and TIR-domain-containing adapter-inducing interferon-*β* (TRIF) pathways, we also tested the effect of monophosphoryl lipid A (MPLA), an TLR4 adjuvant associated with TRIF-biased signaling MPLA, which exhibits low toxicity and is currently licensed to human vaccines [20, 21]. In the present study, the parameters used to monitor Th2 activities were serum levels of IgE anaphylactic antibodies and the intensity of airway allergic inflammation is shown in a OVA model of asthma [18, 19].

## 2. Material and Methods

### 2.1. Mice

Six- to eight-week-old female C57BL/6 or BALB/c mice were bred at Specific Pathogen-Free Breeding Unit, Institute of Biomedical Sciences (ICB-IV, USP), kept in in ventilated caging system (five animals/cage), and treated according to animal welfare guidelines of ICB (Ethic Protocol 081/09), under National Legislation –11.794 Law 12 h light/dark cycle, food, and water* ad libitum*.

### 2.2. Sensitization and Challenge

Mice were sensitized on days 0 and 7 subcutaneously with 4 *μ*g of ovalbumin (OVA, Sigma-Aldrich, St. Louis, MO, USA) and/or 0.25 *μ*g of Tetanus Toxoid, kindly provided by Dr. Luciana Cerqueira Cezar Leite (Instituto Butantan, SP Brazil), both adsorbed to 1.6 mg of alum gel in 0.2 mL of PBS. OVA without endotoxin contamination (LPS-free OVA) and alum preparation were performed as previously described [18]. Toll-like receptors (TLRs) agonists PolyI:C (Polyinosine-polycytidylic acid), a TLR3 agonist signaling through TIR-domain-containing adapter-inducing interferon-*β* (TRIF) and MPLA (monophosphoryl lipid A), a TLR4 agonist TRIF-biased adjuvant [7, 21] extracted from the rough strain* Salmonella minnesota* R595 (Invivogen, San Diego, CA, USA); and LPS from* Escherichia coli* 055:B5 (Sigma-Aldrich, St. Louis, MO, USA), a TLR4 agonist, were adsorbed onto alum gel. The standard dose of all TLR ligands used was 10 *μ*g. On days 14 and 21, mice were intranasally challenged with 10 *μ*g of OVA or TT in 40 *μ*L of PBS. Sensitization and challenge were done under anesthesia with ketamine (50 mg/kg) and xylazine (20 mg/kg). Animals were euthanized by inhaled halothane 24 h after last challenge; samples were collected, unawares numbered, and decoded after analyses.

### 2.3. Serum Samples and Bronchoalveolar Lavage (BAL)

Blood samples were collected by cardiac puncture, centrifuged, and serum stored at −20°C. BAL was acquired after lung washing with 1 mL of cold PBS via trachea. Total and differential cell counts of BAL fluids were determined by haemocytometer and cytospin preparation stained with Instant-Prov Romanowsky-stain (Newprov, Brazil).

### 2.4. Enzyme-Linked Immunosorbent Assay (ELISA): Serum and BAL

Total mouse IgE was determined by sandwich-ELISA using kit OptEIA ELISA Set (BD, San Diego, USA). OVA-specific IgE was determined by adding serum at multiple dilutions to plates with anti-IgE (SouthernBiotech, Birmingham, AL, USA). After washing, biotin-labelled OVA was added and revealed with avidin-HRP plus substrate. Internal sample arbitrarily assigned as 1000 U was used as standard [18]. OVA-specific IgG1 and IgG2a were measured by coating the plates with 20 *μ*g/mL of OVA. Serum samples were added at multiple dilutions and anti–mouse HRP-IgG1 or -IgG2a (Invitrogen, San Diego, USA) was revealed. Purified mouse IgG1 or IgG2a (Invitrogen) was used as standard. All ELISAs were performed in 96-well maxisorp plates (Nunc, NY, USA). Levels of cytokines in the BAL fluid were assayed by sandwich kit ELISA (BD, San Diego, USA) [22].

### 2.5. Measurement of IgE Anaphylactic Antibodies

The anaphylactic activity of IgE to OVA or TT was evaluated by passive cutaneous anaphylactic reaction (PCA) in rat as previously described [18]. All determinations were made in triplicate and the PCA titers were expressed as the reciprocal of the highest dilution that gave a lesion of >5 mm in diameter. The detection threshold of the technique was established at 1 : 5 dilutions.

### 2.6. Statistical Analysis

Statistical analyses were performed using Graphpad Prism (V.5; GraphPad Software, USA). One- or two-way ANOVA followed by Bonferroni post-test was performed, as appropriate. Differences were considered statistically significant when *P* value ≤0.05. Data was presented as mean ± standard error (SE).

## 3. Results

### 3.1. TLR 4 Agonist Is More Effective Than TLR3 Agonist in Dampening OVA-Induced Th2-Mediated Allergic Responses

**Figure 1:**
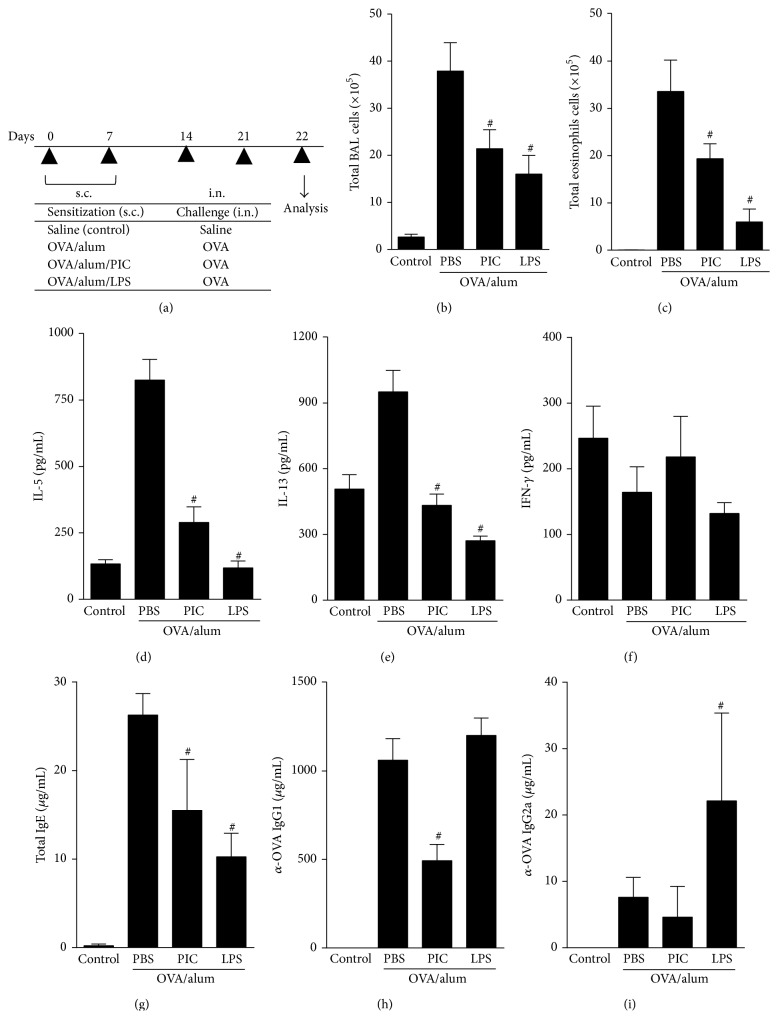
Effects of adsorption of PIC (TLR3) or LPS (TLR4) agonists onto OVA/alum sensitization on OVA-induced cellular and humoral responses. (a) Protocol: C57BL/6 WT mice sensitized with s.c. OVA/alum in the presence or not of PIC (10 *μ*g) or LPS (10 *μ*g) on days 0 and 7 and challenged with OVA i.n. on days 14 and 21. Samples obtained on day 22. (b) Total number of cells and (c) eosinophils in BAL; ((d), (e), and (f)) levels of IL-5, IL-13, and IFN- g in BAL. ((g), (h), and (i)) Levels of serum isotypes: total IgE, OVA-specific IgG1, and OVA-specific IgG2a. Data shown as mean ± SE, one-way ANOVA: ^#^ *P* < 0.05 different from OVA/alum/PBS group (*n* = 5), and experiment was repeated twice.

We have previously shown that TLR4 agonist (LPS) adsorbed to OVA/alum prevented the development of asthma-like responses via MyD88, but not TRIF pathway [18]. In order to ascertain more directly the effect of TRIF signaling, we used the OVA model to compare the effect of Poly I:C, a TLR3 synthetic agonist analog of dsRNA, which signals solely thorough TRIF; with LPS, a TLR4 agonist that signals thorough MyD88 and TRIF pathways. For this, BALB/c mice were sensitized to OVA adsorbed to alum in the absence (allergic group) or presence of agonists of TLR3 (Poly-I:C) or TLR4 (LPS). Overall, both TLRs agonists when adsorbed to OVA/alum dampened Th2 responses when compared to allergic (OVA/alum) group (Figure 1). However, LPS was consistently more effective than PIC in decreasing total cell counts and eosinophil number in BAL fluid (Figures 1(b)-1(c)), IL-5, and IL-13 production (Figures 1(d)-1(e)), and IgE levels (Figure 1(g)). Importantly, the levels of IFN*γ* in BAL in PIC or LPS groups were not increased and were similar to naive or allergic (PBS) groups (Figure 1(f)). Regarding antibody production, again LPS was more effective than PIC in decreasing IgE (Figure 1(g)). PIC but not LPS decreased OVA-specific IgG1 isotype (Figure 1(h)) while LPS increased IgG2a production (Figure 1(i)). Altogether, these results indicate that LPS was more efficient than PIC in inhibiting Th2-mediated airway allergic response.

### 3.2. LPS Is More Effective Than MPLA in Dampening Toxoid-Induced Th2-Mediated Allergic Responses

**Figure 2:**
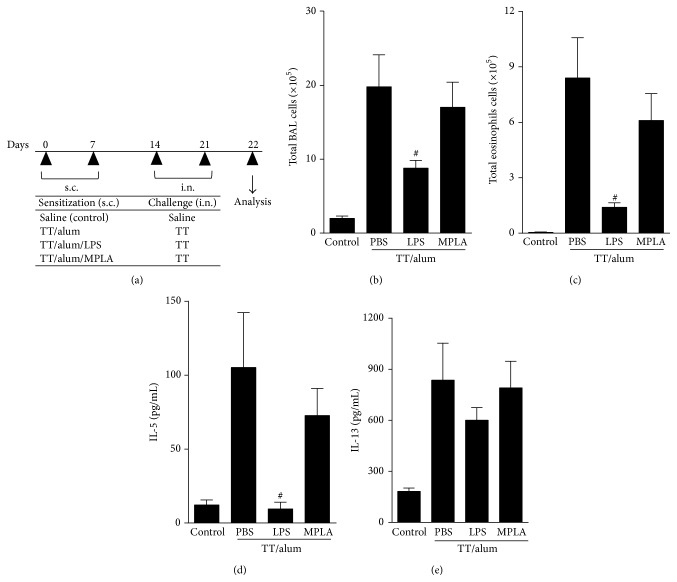
Effects of adsorption of LPS or MPLA onto TT/alum sensitization on TT-mediated airway allergic responses. (a) Protocol: BALB/c mice sensitized with s.c. TT/alum in the presence or not of LPS (10 *μ*g) or MPLA (10 *μ*g) on days 0 and 7, and challenged with TT i.n. on days 14 and 21. Samples obtained on day 22: (b) total number of cells and (c) eosinophils in BAL; ((d) and (e)) levels of IL-5 and IL-13 in BAL. Data shown as mean ± SE, one-way ANOVA: ^#^ *P* < 0.05; different from TT/alum/PBS group (*n* = 5), and experiment was repeated twice.

**Figure 3:**
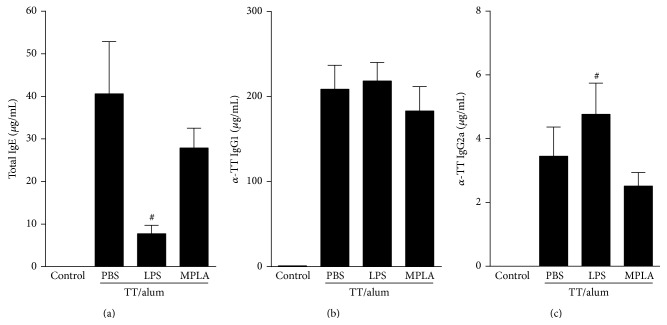
Effects of adsorption of LPS or MPLA onto TT/alum sensitization on serum isotype production. Protocol as shown in Figure 2(a). Concentration of serum isotypes: (a) total IgE, (b) TT-specific IgG1, and (c) TT-specific IgG2a. Data shown as mean ± SE, one-way ANOVA: ^#^ *P* < 0.05; different from TT/alum/PBS group (*n* = 5), and experiment was repeated twice.

We next adapted the OVA model protocol to tetanus toxoid antigen. As depicted in Figure 2(a), sensitizations of tetanus toxoid adsorbed to alum (TT/alum group) followed by i.n. challenges resulted in airway allergic inflammation, as revealed by increased total cell counts of inflammatory cells in BAL fluid, constituted mainly of eosinophils when compared to control group (Figures 2(b)-2(c)). We also found in TT/alum group an increased level of type 2 cytokines (IL-5 and IL-13) in BAL when compared to control group (Figures 2(d)-2(e)) thus, confirming the development of airway allergic inflammation. Having established in the OVA model the role of MyD88 and TRIF signaling in the prevention of allergic responses, we now tested the effects of absorbing two different preparations of LPS/lipid A onto tetanus toxoid/alum. One preparation was obtained from* Escherichia coli* 055:B5 that signals thorough TLR4 via MyD88 and TRIF pathways and other designated MPLA, which is a TRIF-biased TLR4 agonist [21]. As shown in Figure 2, the addition of LPS to tetanus toxoid alum preparation inhibited significantly the development allergic airway inflammation, as evidenced by lower number of total cell counts and eosinophils in BAL compared to TT/alum group (Figures 2(b)-2(c)). Also IL-5, but not IL-13, levels in BAL were significantly decreased in LPS group (Figures 2(d)-2(e)). In contrast, although Th2 responses of MPLA group were lower than TT/alum group, these responses did not reach statistical significance (Figure 2). We conclude tetanus toxoid adsorbed to alum behaves like an allergen and that LPS, but not MPLA, efficiently dampens alum pro-Th2 activity. To confirm this, we determined systemic antibody production by measuring serum levels of total IgE. As shown in Figure 3, sensitization and challenge with tetanus toxoid increased IgE levels when compared to control group. The addition of LPS, but not MPLA, to alum decreased significantly IgE levels (Figure 3(a)). Conversely, IgG1 antibodies against tetanus toxoid were similar in PBS, LPS, or MPLA groups (Figure 3(b)) while IgG2a specific antibodies were increased in LPS when compared to PBS group (Figure 3(c)). These results indicate that LPS prevented the production of IgE anaphylactic antibodies, did not interfere significantly in TT-specific IgG1, and augmented TT-specific IgG2a antibody production.

### 3.3. LPS Potentiate Anti-Toxoid Antibody Production in a Model with Two Antigens

**Figure 4:**
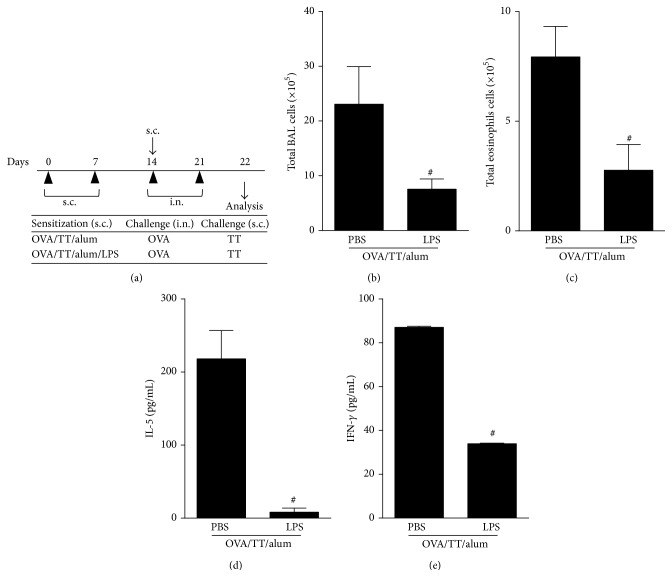
Effects of adsorption of LPS onto OVA/TT/alum sensitization on OVA-induced airway allergic inflammation. (a) Protocol: BALB/c mice sensitized with s.c. OVA/TT/alum in the presence or not of LPS (10 *μ*g) on days 0 and 7 and challenged with i.n. OVA on days 14 and 21. Samples obtained on day 22. (b) Total number of cells and (c) eosinophils in BAL: ((d) and (e)) levels of IL-5 and IFN-*γ* in BAL. Data shown as mean ± SE, one-way ANOVA: ^#^ *P* < 0.05; different from OVA/TT/alum/PBS group (*n* = 5), and experiment was repeated twice.

**Figure 5:**
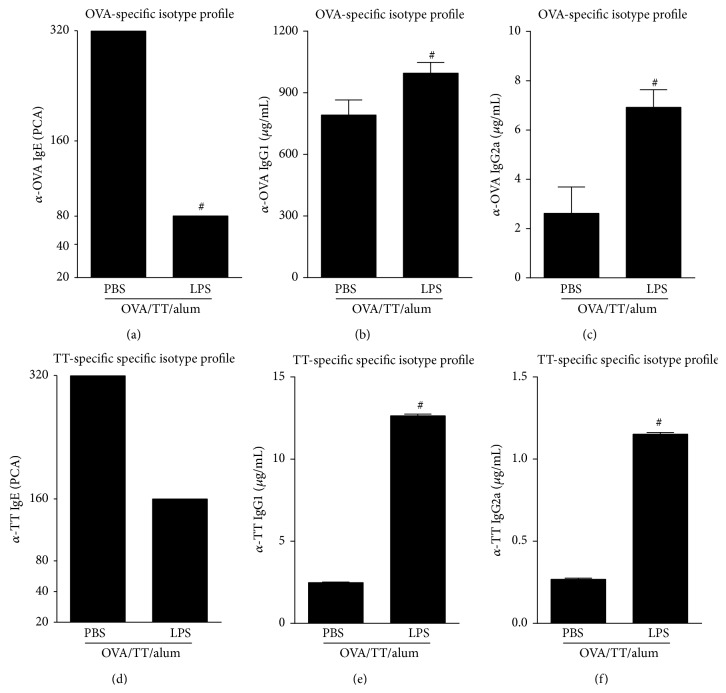
Effects of adsorption of LPS onto OVA/TT/alum sensitization on OVA- or TT-induced isotype production. Protocol shown in Figure 4(a). ((a) and (c)) Concentration of OVA-specific isotypes: (a) PCA titers anaphylactic IgE, (b) IgG1, and (c) IgG2a. ((d) and (f)) Concentration of TT-specific isotypes: (d) PCA levels of anaphylactic IgE, (e) IgG1, and (c) IgG2a. Data shown as mean ± SE, one-way ANOVA: ^#^ *P* < 0.05; different from OVA/TT/alum/PBS group (*n* = 5), and experiment was repeated twice.

The benefits of combined vaccines such as diphtheria, pertussis, tetanus (DPT) vaccine are multiple including fewer injections and lower cost. However, special attention must be taken with the immunogenicity of individual antigens when combining unrelated antigens since it can be impaired, enhanced, or not affected [23, 24]. To test antigen combination in our model, we used OVA and TT antigens as unrelated antigens, adsorbing them to alum in the presence or absence of LPS, as depicted in Figure 4(a). To monitor Th2 activities we determined OVA-specific responses such as airway allergic inflammation and serum titers of anaphylactic IgE after i.n. OVA challenge. TT-specific responses were determined by serum isotype antibody production after TT s.c challenge. Regarding OVA-specific airway allergic responses, we found that addition of LPS inhibited total cells and eosinophil numbers recruitment to BAL when compared to (PBS) allergic group (Figures 4(b)-4(c)). In addition, IL-5 and, of note, IFN*γ* levels in BAL were also decreased in LPS group as compared to PBS group (Figures 4(d)-4(e)), indicating that LPS do not shift towards an OVA-induced lung Th1 response. As expected, LPS blocked OVA-specific IgE anaphylactic antibodies as revealed by passive cutaneous anaphylaxis titers (Figure 5(a)), thus confirming the antiallergic effect of LPS in our model. In addition, OVA-specific IgG1 and IgG2a antibodies were increased in LPS group compared to PBS group (Figures 5(b)-5(c)). Regarding TT-specific responses, LPS addition showed a similar effect to that obtained with OVA-specific responses (Figures 5(d)–5(f)). Of note, we found that the concentrations of TT-specific antibodies (IgG1 and IgG2a) were lower (Figures 5(e)-5(f)) than those obtained when sensitization and challenge were done with TT alone (Figures 3(b)-3(c)), indicating that the phenomenon known as antigenic completion has occurred [24]. Nevertheless and, notably, addition of LPS to OVA/TT/alum resulted in a higher production of IgG1 and IgG2a anti-TT antibodies when compared to TT/OVA/alum group. As expected, TT-specific IgE titers were lower in LPS group when compared with PBS group (Figure 5(d)). Therefore, the addition of LPS to alum-based vaccine with two unrelated antigens was able to increase IgG1 and IgG2a antibody production and decrease anaphylactic IgE titers, a more desirable profile for neutralizing antibodies.

## 4. Discussion

Historically, vaccines have been developed and employed to protect humans or animals against infectious diseases [25]. Alum adjuvant has been empirically used in human vaccination to apparently boost neutralizing antibodies against toxins (DT vaccine) or viruses (hepatitis B) [17]. In contrast, TLRs agonists are viewed as adjuvants that usually favor cellular immunity and the development of Th1 or Th17 cells [26]. Although alum has been used extensively, its pro-Th2 activity, as revealed in animal models of allergies, might limit its efficacy in vaccination against infectious pathogens. We have previously shown that LPS adsorbed onto alum dampened Th2 responses via MyD88 pathway and modulated antibody isotype pattern by increasing IgG2a (Th1) and decreasing IgE (Th2) antibodies [18]. Here we extended our study to tetanus toxoid antigen and tested LPS as well as MPLA, a TLR4 adjuvant associated with a bias toward TRIF signaling [21]. Because TRIF signaling results in type I IFNs production, which are known to exert an inhibitory activity on Th2-mediated responses [26–28], we first investigated, in the OVA model, the effect of signaling via TRIF pathway using PIC, a unique TLR3 agonist that signals solely through TRIF pathway. Indeed, we found that signaling through TRIF pathway could inhibit some Th2-mediated responses. However, LPS that signals through MyD88 and TRIF pathways was, by far, more effective than PIC in dampening all tested Th2 associated responses. Even tough, we were still interested in comparing the effect of MPLA, a TRIF-biased adjuvant licensed to human vaccination [21] with LPS using TT, an antigen used in human vaccination. We found that sensitization with TT/alum, as occurred with OVA, induces a strong TT-mediated airway allergic inflammation confirming the pro-Th2 activity of alum and indicating that TT is a good allergen. The Th2-associated airway allergic responses were significantly blocked by LPS, but not by MPLA. More importantly, regarding antibody production, LPS but not MPLA inhibited significantly total IgE production while maintaining the levels of IgG1 or IgG2a anti-TT. Therefore, it is clear that absorption of LPS to alum blocks its pro-Th2 activities that are undesirable because they might reduce anti-infectious immunity. Another fact highlighted in our study is related to antigenic competition. We found that the antibody response to TT was higher when mice were sensitized solely with TT than when sensitized with OVA plus TT. Indeed, when designing vaccine with multiple antigens it is important to test whether the combination does not decrease the immunogenicity of single components. This was the case of a multivalent foot rot vaccine [29]. Also, inactivated poliovirus vaccine combined with diphtheria and TT and whole cell pertussis vaccine diminished antibody response to pertussis vaccine [30]. Conversely, whole cell pertussis vaccine combined with diphtheria toxoid boosted the immune response to individual components [31, 32]. In our model, although the combination of OVA plus TT decreased TT-specific antibodies indicating antigenic completion, the addition of LPS potentiates IgG1 and IgG2a anti-TT antibodies while decreasing IgE antibodies revealing that LPS boost the production of anti-infectious antibodies. We believe that LPS acts during the sensitization phase impairing Th2 priming. Supporting this assumption it has been shown that TLR ligands can activate dendritic cells to release an uncharacterized MyD88-dependent negative signal that acts on Th cells and impairs Th2 cell development [33]. Also, it is possible that the efficiency of antigen presentation by dendritic cells is dependent on the presence of TLR ligands and that the generation of peptide–MHC class II complexes is controlled by TLRs in the phagosome as shown by Blander and Medzhitov [34].

The high toxicity of LPS precludes its use in humans, but synthetic TLR4 agonists, with low toxicity, have been developed and we attested their antiallergic effect in the OVA model [18]. In addition, other TLRs agonists can be used. For instance, subcutaneous administration of alum vaccine of ragweed-TLR9 agonist was clinically effective in the treatment of allergic rhinitis [35]. In addition, treatment with a novel TLR9 agonist showed clinical efficacy in persistent allergic asthma [36].

## 5. Conclusions

Our work highlights that the pro-Th2 properties of alum adjuvant can be suppressed by the absorption of LPS indicating the potential use of TLR agonists in alum-based vaccines against a variety of antigens in which antibody production is essential for the immune therapeutic effects and where the alum pro-Th2 activities are detrimental. Our data also show that it is possible to block the development of Th2-mediated responses by TLRs agonists without deviation to a Th1 polarized phenotype.
